# Indole Diketopiperazine Alkaloids from the Marine Sediment-Derived Fungus *Aspergillus chevalieri* against Pancreatic Ductal Adenocarcinoma

**DOI:** 10.3390/md22010005

**Published:** 2023-12-20

**Authors:** Dina H. El-Kashef, Deborah D. Obidake, Katja Schiedlauske, Alina Deipenbrock, Sebastian Scharf, Hao Wang, Daniela Naumann, Daniel Friedrich, Simone Miljanovic, Takin Haj Hassani Sohi, Christoph Janiak, Klaus Pfeffer, Nicole Teusch

**Affiliations:** 1 Institute of Pharmaceutical Biology and Biotechnology, Heinrich Heine University Düsseldorf, 40225 Düsseldorf, Germany; dihas102@hhu.de (D.H.E.-K.);; 2 Department of Pharmacognosy, Faculty of Pharmacy, Minia University, 61519 Minia, Egypt; 3 Institute of Medical Microbiology and Hospital Hygiene, Medical Faculty, Heinrich Heine University, 40225 Düsseldorf, Germany; 4 Hainan Key Laboratory for Research and Development of Natural Products from Li Folk Medicine, Institute of Tropical Bioscience and Biotechnology, Chinese Academy of Tropical Agricultural Sciences, Haikou 571101, China; 5 Department of Chemistry and Biochemistry, University of Cologne, 50939 Cologne, Germany; 6 Institute of Inorganic Chemistry and Structural Chemistry, Heinrich Heine University Düsseldorf, 40225 Düsseldorf, Germany

**Keywords:** *Aspergillus chevalieri*, indole diketopiperazine alkaloids, prenylation, genome sequencing, X-ray diffraction, pancreatic cancer, pancreatic ductal adenocarcinoma

## Abstract

A new prenylated indole diketopiperazine alkaloid, rubrumline P (**1**), was isolated along with six more analogues and characterized from the fermentation culture of a marine sediment-derived fungus, *Aspergillus chevalieri*, collected at a depth of 15 m near the lighthouse in Dahab, Red Sea, Egypt. In the current study, a bioassay-guided fractionation allowed for the identification of an active fraction displaying significant cytotoxic activity against the human pancreatic adenocarcinoma cell line PANC-1 from the EtOAc extract of the investigated fungus compared to the standard paclitaxel. The structures of the isolated compounds from the active fraction were established using 1D/2D NMR spectroscopy and mass spectrometry, together with comparisons with the literature. The absolute configuration of the obtained indole diketopiperazines was established based on single-crystal X-ray diffraction analyses of rubrumline I (**2**) and comparisons of optical rotations and NMR data, as well as on biogenetic considerations. Genome sequencing indicated the formation of prenyltransferases, which was subsequently confirmed by the isolation of mono-, di-, tri-, and tetraprenylated compounds. Compounds rubrumline P (**1**) and neoechinulin D (**4**) confirmed preferential cytotoxic activity against PANC-1 cancer cells with IC_50_ values of 25.8 and 23.4 µM, respectively. Although the underlying mechanism-of-action remains elusive in this study, cell cycle analysis indicated a slight increase in the sub-G1 peak after treatment with compounds **1** and **4**.

## 1. Introduction

Pancreatic ductal adenocarcinoma (PDAC), the most common form of pancreatic neoplasm, is considered the fourth leading cause of cancer-related mortality worldwide due to its high aggressiveness and malignancy with a poor survival rate [1,2]. Despite breakthroughs in diagnostic tools, surgical treatments, and chemotherapy, the overall survival rates of PDAC remain low, and the only available therapies are classical chemotherapeutics with an unselective cytotoxic profile. Consequently, there is an immense need to find new drugs or drug leads for curing this disease [2,3]. Many natural products have been reported to exert anticancer effects against pancreatic cancer [3,4,5]. As part of our ongoing search for discovering novel potential drug candidates for use against PDAC that originate from a natural source [6], a bioassay-guided isolation of secondary metabolites was conducted on a marine sediment-derived fungus, *Aspergillus chevalieri*, collected from the Red Sea at a depth of 15 m close to the lighthouse of Dahab, Egypt. Accordingly, seven prenylated indole diketopiperazine alkaloids, including a rare tetraprenylated derivative that has been reported only once before, were isolated from the bio-active fraction, identified, and further pharmacologically characterized [7,8]. Indole diketopiperazine alkaloids (DKPs) represent a group of secondary metabolites broadly distributed in filamentous fungi, especially in the genera *Aspergillus* and *Penicillium* [9]. Biosynthetically, in fungi, the core cyclic dipeptide nucleus of DKPs is mostly originated from the condensation of two tryptophan amino acids or one tryptophan molecule and another amino acid, assembled by non-ribosomal peptide synthetases (NRPSs) [8,10]. Different putative tailoring enzymes contribute to the structural diversity of DKP scaffolds, including oxidoreductases, hydrolases, methyl transferases, prenyltransferases, and ligases [11,12]. Prenylated indole diketopiperazines are constituted of an indole diketopiperazine backbone and isoprenoid moieties, in which prenylation is catalyzed by prenyltransferases acting as tailoring enzymes [7,8,13]. The subsequent prenylation cascade of echinulin and neoechinulin series of alkaloids in *Aspergillus ruber* has been demonstrated to be mediated via two prenyltransferases, EchPT1 and EchPT2. These two enzymes are involved in the prenylation and reverse prenylation cascades taking place at different positions of the indole DKP core structure of echinulin and neoechinulin congeners, resulting in the formation of mono-, di-, tri-, and tetraprenylated derivatives [7,8]. Indole DKPs are not only characterized by the diversities in their chemical structures but are also reported to exhibit significant biological activities, such as anti-inflammatory, anticancer, antiviral, anti-neurogenerative, and antioxidant activities [9,14]. Various indole DKPs have been evaluated for their anticancer activities against several cancer lines such as HL-60, P388, BEL-7402, A-549, PC12, and HeLa cells via induction of apoptosis and/inhibition of cell proliferation [14,15,16,17,18]. Owing to their improved lipophilicity, prenylated natural products are proven to possess better binding affinity to target proteins and better bioavailability compared to nonprenylated derivatives, creating a direct impact on the biological activity [19,20]. Therefore, prenylated DKPs have demonstrated to be possible candidates for drug development and discovery [21,22]. In the current study, we report the isolation of different prenylated indole diketopiperazine alkaloids, including a new triprenylated derivative rubrumline P (**1**) and a rare tetraprenylated known congener 1Q2 (**7**), from a marine sediment-derived fungus, *A. chevalieri*, through a bioassay-guided fractionation. In addition, the crystal structure of a known derivative, rubrumline I (**2**), is reported for the first time. Genome sequencing of the fungus revealed secondary metabolite regions presumably involved in the synthesis of the reported prenylated metabolites. Furthermore, an analysis of the isolated compounds’ cytotoxic efficacy against pancreatic cancer cell line PANC-1 cells has been performed, followed by cell cycle analyses of the isolated compounds.

## 2. Results and Discussion

### 2.1. Bioactivity-Guided Fractionation of the Ethyl Acetate Extract of Aspergillus chevalieri

**Figure 1:**
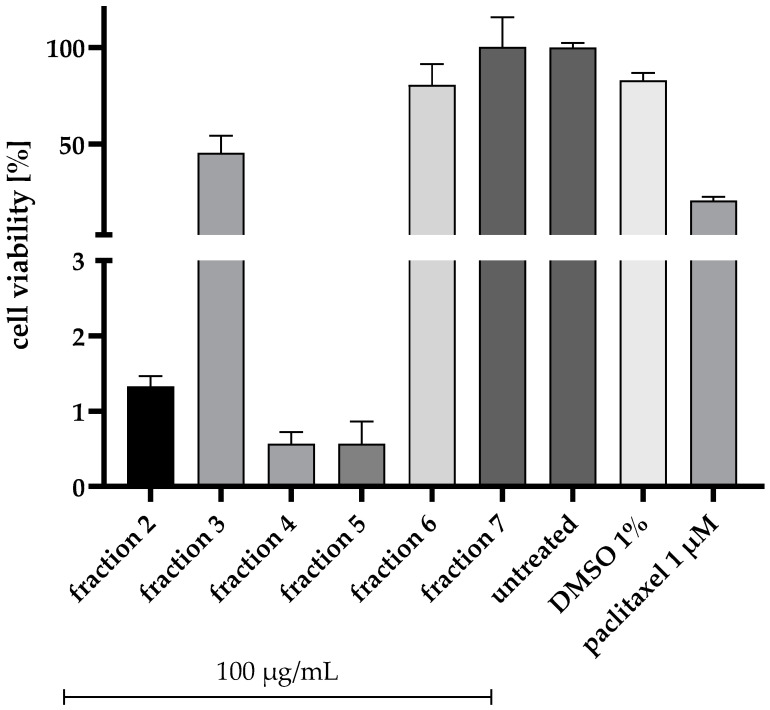
Results of the bioassay-guided fractionation: Cytotoxic effects of the vacuum liquid chromatography (VLC) fractions of *Aspergillus chevalieri* on PANC-1 cells. Cells were seeded into a 96-well plate at a concentration of 5 × 10^3^ cells/well and treated for 72 h after seeding with the different VLC fractions at a concentration of 100 μg/mL. A solvent control (1% DMSO) and a positive control (1 μM paclitaxel) were incubated for 72 h. Cell viability is expressed as the percentage of untreated cells. Error bars indicate standard errors of the mean (SEM) (*n* = 1 with three technical replicates per subjected fraction).

After cultivating *A. chevalieri* on a solid rice medium supplemented with 3.5% NaCl (mimicking the salinity of seawater), the crude ethyl acetate extract of the fungus was initially subjected to chromatographic separation via vacuum liquid chromatography. Subsequently, the produced VLC fractions were preliminary tested for their cytotoxic potential against the human pancreatic PANC-1 cancer cell line at a concentration of 100 µg/mL. Remarkable cytotoxic activity was observed for fractions 4 and 5 after 72 h compared to 1 µM of the microtubule inhibitor paclitaxel, part of the first-line treatment regimen for PDAC patients (Figure 1). Incubation of fraction 4 for 72 h reduced the viability of PANC-1 cells by about 99%. A comparable result was observed for fraction 5. Based on the bioassay result and HPLC profile of fraction 4 (100% EtOAc) compared to those of fraction 5 (90% DCM-MeOH), which was an oily fraction, fraction 4 was selected for further chromatographic separations to isolate and identify the active compounds.

### 2.2. Structure Elucidation

**Figure 2:**
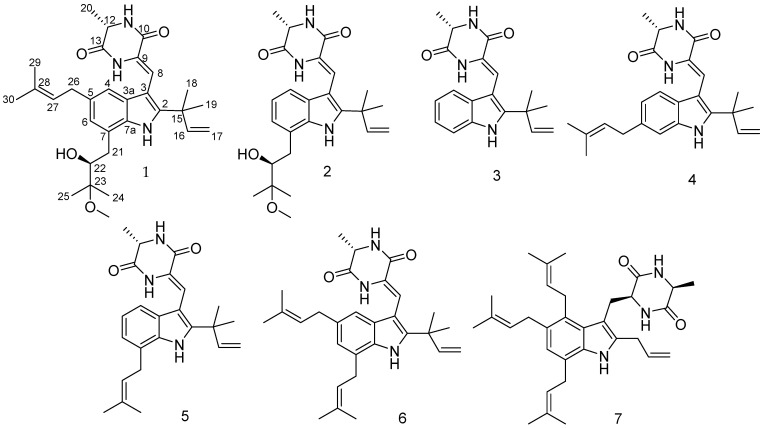
Structures of the isolated compounds from *Aspergillus chevalieri*.

After our chromatographic fractionation of the crude extract of *A. chevalieri* on VLC, various separations were conducted on other stationary phases, followed by final purifications utilizing semi-preparative HPLC affording seven indole diketopiperazine alkaloids, compounds **1**–**7** (Figure 2), including one new alkaloid (**1**) in addition to six known mono-, di-, tri-, and tetraprenylated analogues identified as rubrumline I (**2**) [23], neoechinulin A (**3**) [24], neoechinulin D (**4**) [25], variecolorin G (**5**) [26], dehydroechinulin (**6**) [27], and 1Q2 (**7**) [8] through comparison of their 1D/2D NMR spectroscopic data with those in the literature.

**Figure 3:**
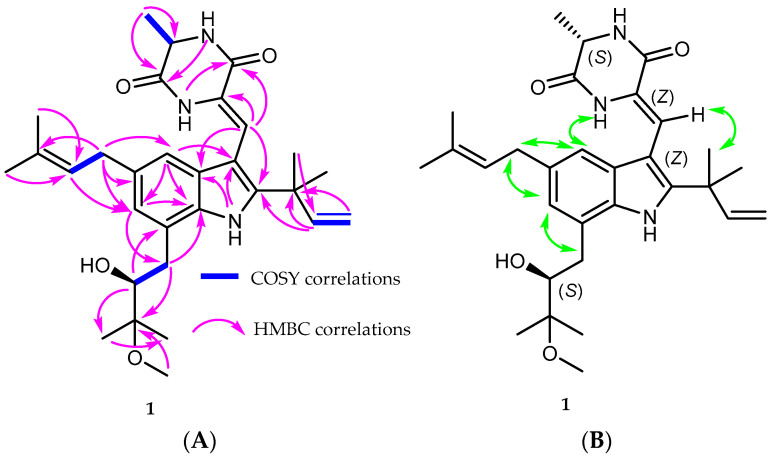
(**A**) Key ^1^H-^1^H COSY and HMBC correlations of compound **1**. (**B**) Key NOESY correlations of compound **1**.

**Figure 4:**
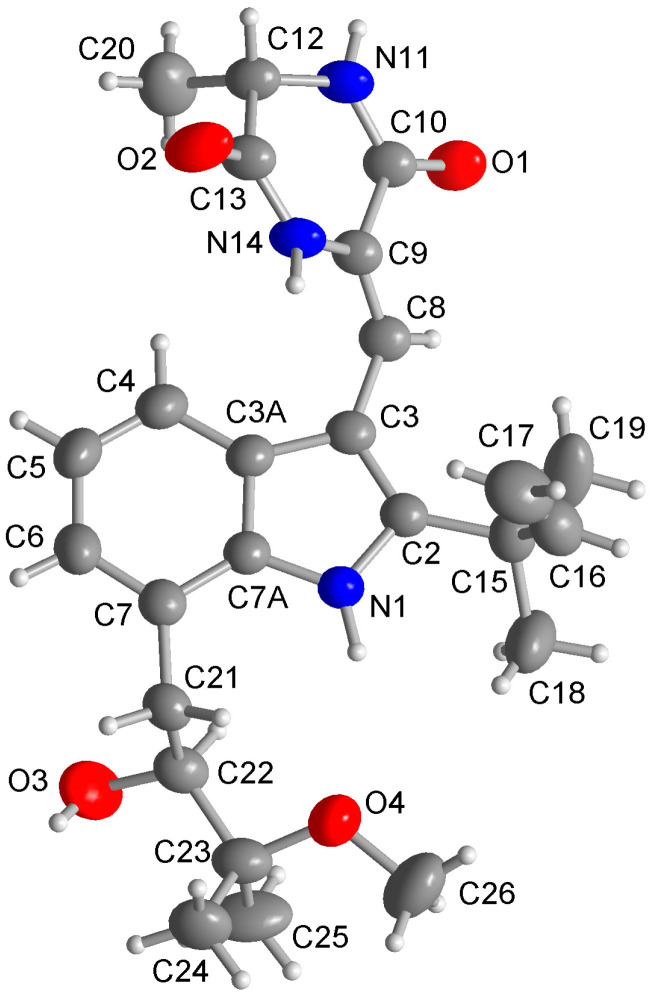
Molecular structure of **2** from a single-crystal X-ray structure determination analysis (50% thermal ellipsoids, H atoms with arbitrary radii). See Figure S9 in the Supplementary Materials for the packing diagram and a diagram of the hydrogen-bonding network.

Compound **1** was isolated as a beige-brown residue. Its molecular formula was determined to be C_30_H_41_N_3_O_4_ based on a positive HRESIMS analysis, indicating 12 degrees of unsaturation. The ^1^H-NMR data (Table 1) and HSQC analysis exhibited signals for two meta-coupled aromatic protons (*δ*_H_ 6.93, d, *J* = 1.5 Hz, H-4; and *δ*_H_ 6.74, d, *J* = 1.5 Hz, H-6), six singlet methyls at *δ*_H_ 1.51, 1.50, 1.28, 1.23, 1.72, and 1.71, assigned for H_3_-18, H_3_-19, H_3_-24, H_3_-25, H_3_-29, and H_3_-30, respectively, a doublet methyl (*δ*_H_ 1.60, d, *J* = 7.0, H_3_-20), one methoxy group (*δ*_H_ 3.28, 23-OCH_3_), and three protons of a vinyl group (*δ*_H_ 6.05, dd, *J* = 17.4, 10.5 Hz, H-16; *δ*_H_ 5.14, d, *J* = 10.5 Hz, H-17a; and *δ*_H_ 5.12, d, *J* = 17.4 Hz, H-17b). Moreover, proton signals for two methylenes (H-21 and H-26), four methines—including one aliphatic proton (*δ*_H_ 4.27, q, *J* = 6.9 Hz; H-12), two olefinic protons (*δ*_H_ 7.23, s, H-8; and *δ*_H_ 5.35, m, H-27), and one oxygenated proton (*δ*_H_ 3.80, d, *J* = 8.7 Hz, H-22)—and three NH protons (NH-1, NH-11, and NH-14) were also detected. The APT and HMBC spectroscopic data (Table 1) assigned the presence of 30 carbons, allocated for: seven methyl groups and one methoxy group (CH_3_-18, CH_3_-19, CH_3_-20, CH_3_-24, CH_3_-25, CH_3_-29, CH_3_-30, and 23-OCH_3_); three methylenes, including one olefinic (CH_2_-17) and two aliphatic (CH_2_-21, and CH_2_-26) groups; seven methines, with five olefinic/aromatic carbons (CH-4, CH-6, CH-8, CH-16, and CH-27), one oxygenated (CH-22), and one aliphatic (CH-12); and twelve quaternary carbons, accounting for two carbonyls (C-10, and C-13), eight olefinic/aromatic carbons (C-2, C-3, C-3a, C-5, C-7, C-7a, C-9, and C-28), one oxygenated (C-23), and one aliphatic carbon (C-15). Detailed inspection of the 1D and 2D NMR spectra of compound **1** revealed that compound **1** is an indole diketopiperazine derivative that is structurally similar to the known co-isolated rubrumline I (**2**), which was previously only characterized once from *Eurotium rubrum* F33, a marine sediment-derived fungus collected at a depth of 2067 m under the South Atlantic Ocean [23]. However, the signal for a methine group resonating at *δ*_H_ 7.06/*δ*_C_ 117.3 (CH-5) in rubrumline I was replaced by signals for an isoprenyl moiety in **1**. This assignment was suggested from the molecular composition of **1** (C_30_H_41_N_3_O_4_), which, compared to rubrumline I (2) (C_25_H_32_N_3_O_4_), bears an additional isoprenyl group (-C_5_H_9_) constituting the last remaining degree of unsaturation. Moreover, this assignment was further deduced based on COSY correlations between H_2_-26 and H-27, together with the HMBC correlations from H_2_-26 to C-4 (*δ*_C_ 116.6), C-5 (*δ*_C_ 134.5), C-6 (*δ*_C_ 123.8), and C-28 (*δ*_C_ 132.0) and from H_3_-29/H_3_-30 to C-28, and C-27 (*δ*_C_ 124.3) (Figure 3A), together with the NOE correlations between H_2_-26 and H-4, and between H_2_-26 and H-6. The geometry of the double bond at C-8 was assigned to be *Z* based on the NOESY correlations between H-4 and NH-14 and between H-8 and CH_3_-18, together with the absence of cross peaks from H-8 to NH-14, which suggests the stable configuration of compound **1** (Figure 3B) that is also identical to the X-ray diffraction analysis structure of compound **2** (Figure 4). Moreover, the downfield shift of the olefinic proton H-8 (δ_H_ 7.23) implies a deshielding effect of the 10- carbonyl group, confirming the *Z* configuration of the double bond at C-8 [23,26,28]. Therefore, compound **1** was elucidated as a 5-prenyl analog of compound **2**, representing a new rubrumline derivative, for which the trivial name rubrumline P is proposed.

The specific optical rotation of compound **1** is negative, which is consistent with the negative values reported for other known neoechinulin A series of indole diketopiperazine alkaloids with 12*S* and 22*S* configurations, proposing that the new compound **1** shares the same absolute configuration as the known derivatives [23,24,29]. Although the absolute configuration of the previously reported rubrumline derivatives was assigned using a modified Mosher’s method and CD measurement, no crystal structure has been reported so far for these derivatives [23]. Herein, we present an independent assignment of the absolute configuration of the known rubrumline I (**2**) via single-crystal X-ray diffraction analysis through anomalous dispersion for the first time, allowing an unambiguous assignment of the absolute configuration of **2** as 12*S* and 22*S* (Figure 4). Based on NMR data, optical rotations and biogenetic considerations, the same absolute configurations, as elucidated for compound **2** via X-ray diffraction analysis, can be assumed for the other alkaloids as well.

### 2.3. Genome Sequencing

The genome assembly of *Aspergillus chevalieri* resulted in a total length of 29,419,542 bp, with an N50 fragment length of 1,488,407 bp and an average coverage of 3960×. The assembly consists of 192 contigs, of which 8 contigs contain more than 1,000,000 base pairs (Table 2). These eight contigs correspond to the eight chromosomes of the fungal genome as represented in the reference strain in the NCBI database (NCBI RefSeq assembly: GCF_016861735.1) [30].

**Figure 5:**
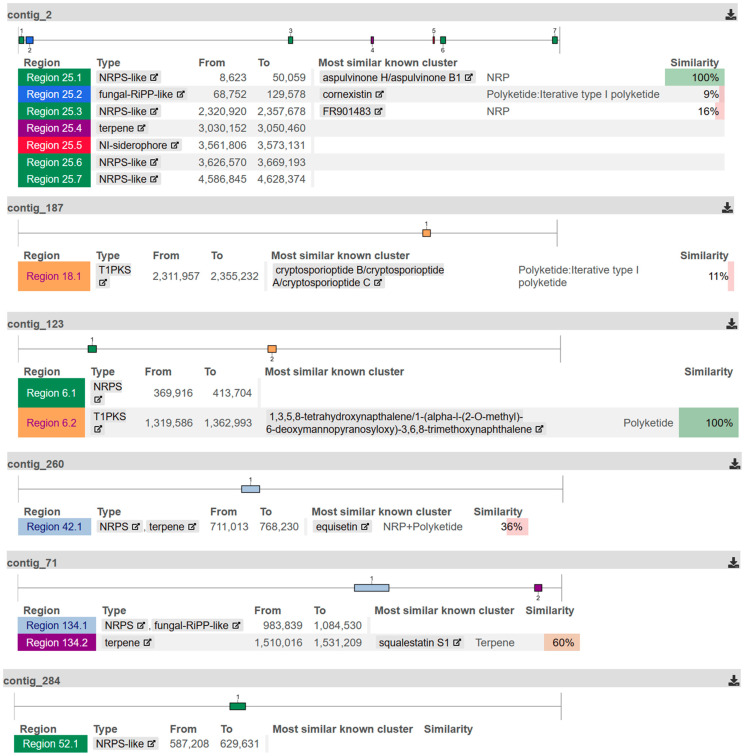
Identified secondary metabolites regions for *Aspergillus chevalieri*. For each of the six genome assembly contigs on which regions for secondary metabolites were identified, the position of the region and the structure type of the metabolites encoded there are shown. In addition, the most similar metabolite cluster already known is indicated with the associated cluster type and the degree of similarity (%).

Regions coding for potential secondary metabolites were found on six of these eight contigs (Figure 5). Most were gene clusters for non-ribosomal peptide synthetases (NRPSs), which were found on five contigs, followed by regions for type I polyketide synthases (T1PKSs), terpenes, and fungal ribosomally synthesized and post-translationally modified peptide (RiPP)-like secondary metabolites, on two contigs each. Regions for NI-siderophones were also found on one contig. In an additional analysis with InterProScan, a potential region for terpenoid cyclases and protein prenyltransferases was also found on a 21,917 bp-long contig, which does not belong to the core genome.

Analysis of the gene clusters in the genome of *A. chevalieri* revealed the presence of gene regions for the production of non-ribosomal peptide synthetases, polyketide synthases, and terpenes. There is also an evidence that this fungus is able to synthesize prenyltransferases. Both findings indicate that *A. chevalieri* has the ability to synthesize isoprenylated natural products. The synthesis of isoprenylated natural products through the formation of echinulin prenyltransferase 1 (EchPT1) has so far only been described for other members of the genus *Aspergillus* (*A. fumigatus*, *A. oryzae*, and *A. ruber*), but not yet for *Aspergillus chevalieri* [8,31,32].

### 2.4. Cytotoxicity and Cell Cycle Analyses in the Pancreatic Ductal Adenocarcinoma Cell Line PANC-1

Based on the remarkable cytotoxicity results from the bioactivity-guided screening of the starting fraction 4, the purified compounds **1** and **4** were characterized in full dose–response curves in the selected pancreatic cancer cell line. The respective cytotoxic efficacy levels are displayed in Table 3.

In contrast to the standard-of-care treatment paclitaxel, for which IC_50_ values in the low nanomolar range are reported [33], compounds **1** and **4** display only moderate cytotoxic efficacy levels, with IC_50_ values of 25.8 and 23.4 µM, respectively.

**Figure 6:**
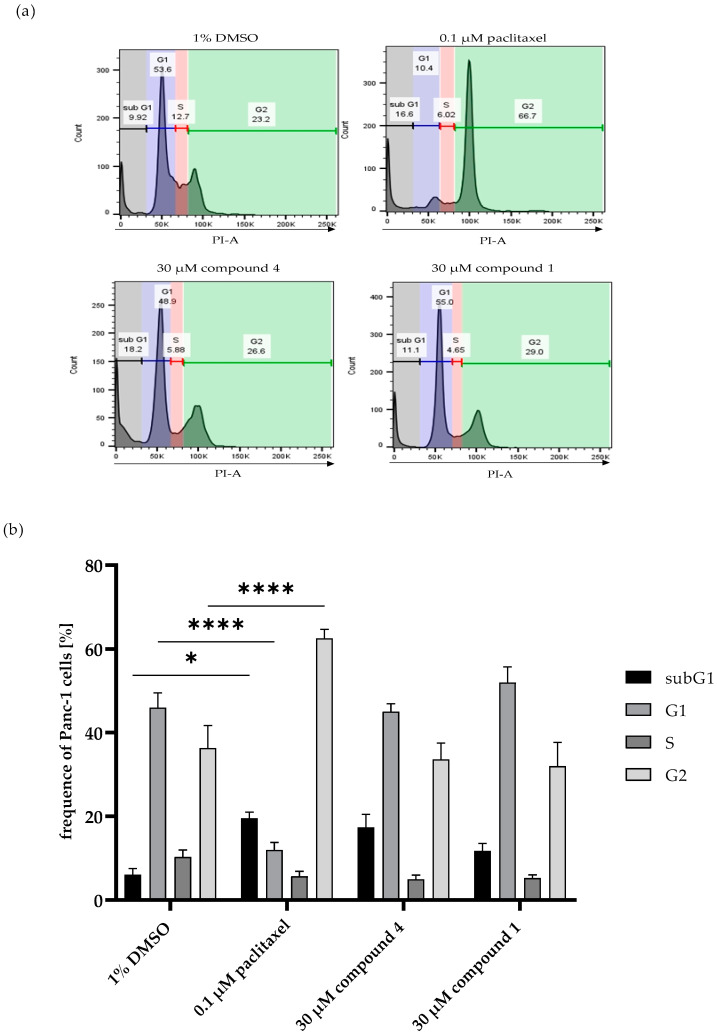
Detection of cell cycle phases in PANC-1 cells. The cells were treated with 30 µM of compound 1, 30 µM of compound 4, or 0.1 µM paclitaxel for 24 h. Cell cycle analysis was performed according to the Nicoletti method. (**a**) Percentage of PANC-1 cells in the different cell cycles after 24 h of treatment with compound 1, compound 4, or paclitaxel, shown in explementary histograms. (**b**) Quantification of cell cycle phases. The cell cycles of at least 10,000 cells were analyzed via flow cytometry. Error bars indicate the standard errors of the mean (*n* = three independent experiments, with * = *p* ≤ 0.05, and **** = *p* ≤ 0.0001).

To determine whether compound **1** or compound **4** affects the cell cycle of PANC-1 cells, a cell cycle analysis was performed. The tumor cells were treated with 30 µM of compound **1** or compound **4** for 24 h, respectively, and stained according to the Nicoletti method. Treatment with the spindle toxin paclitaxel served as a positive control. Treatment with compound **1** or compound **4** did not result in any significant changes in the cell cycle compared to the solvent control DMSO (Figure 6). However, a slight increase in the sub-G1 peak was observed after treatment with compounds **1** and **4**, but this did not reach levels of statistical significance. In contrast, and as expected, treatment with 0.1 µM paclitaxel resulted in the arrest of the cell cycle in the G2 phase and a significant increase in the sub-G1 peak (Figure 6). Therefore, the cytotoxic effects of these compounds might be attributed to inducing apoptosis; however, the exact mechanism-of-action is still elusive.

## 3. Materials and Methods

### 3.1. General Compound Spectroscopic Analysis and Purification Procedures

Optical rotations were determined using a Jasco P-2000 polarimeter. The compounds were dissolved in optically pure solvents Uvasol^®^ (spectroscopic-grade solvents, Merck, Darmstadt, Germany). The 1D and 2D NMR spectra were recorded in CDCl_3_ and MeOD using Bruker Avance III 500 or 600 MHz NMR spectrometers (Bruker BioSpin GmbH, Rheinstetten, Germany). Low-resolution mass spectra (ESI) were measured with an Ion-Trap-API Finnigan LCQ Deca (Thermo Quest, Egelsbach, Germany) mass spectrometer, while high-resolution mass spectrometry (HRESIMS) data were recorded on an FTHRMS-Orbitrap (Thermo-Finnigan, Egelsbach, Germany) mass spectrometer. HPLC analysis was conducted using a Dionex UltiMate-3400 SD with an LPG-3400SD pump coupled to a photodiode array detector (DAD3000RS), with routine detections at 235, 254, 280, and 340 nm. The separation column was a Knauer Eurospher C18 analytical column (125 × 4 mm i.d., 5 m), using the solvent gradient MeOH-(0.1% HCOOH in H_2_O), as follows: 0 min (10% MeOH); 5 min (10% MeOH); 35 min (100% MeOH); 45 min (100% MeOH). Purification of the compounds was performed using semipreparative HPLC on the VWR Hitachi Chromaster HPLC system (5160 pump; 5410 UV detector; Eurosphere-100 C18, 300 mm × 8 mm i.d., 10 m; Knauer, Germany) utilizing MeOH and H_2_O as eluting solvents, employing a flow rate of 5 mL/min. Column chromatography included various stationary phases such as Sephadex LH-20 (0.25–0.1 mm mesh size, Merck) and Merck MN silica gel 60 M (0.04–0.063 mm mesh size). TLC plates precoated with silica gel F254 (Merck) were used for monitoring fractions. Detection of spots on TLC was performed through UV absorption at 254 and 365 nm, or by spraying the plates with an anisaldehyde spray reagent followed by heating.

### 3.2. Fungal Material

The fungus *Aspergillus chevalieri* LH15-100R2 was isolated from a marine sediment, which was collected from the Red Sea in November 2017 at a depth of 15 m near to the lighthouse of Dahab, Egypt. The fungus was identified as *A. chevalieri* (GenBank accession no. OR605556) through amplification and sequencing of the internal transcribed spacer region (ITS), including the 5.8S ribosomal DNA, followed by a BLAST search in NCBI, as described before [34]. This fungal strain is stored at −80 °C in the Institute of Pharmaceutical Biology and Biotechnology, Heinrich Heine University, Düsseldorf.

### 3.3. Fermentation, Extraction, and Isolation

Fermentation of the fungus was carried out in ten 1L Erlenmeyer flasks, each containing 100 g of rice (Oryza Milchreis), 3.5 g of NaCl, and 110 mL of demineralized water. After autoclaving at 121 °C for 20 min then cooling to room temperature, the fungus was transferred to the solid rice medium. The fungal culture was left to grow under static conditions for 23 days at room temperature until the fungus had totally overgrown the medium. Afterwards, the solid fermented fungus was extracted two times with EtOAc (each with 600 mL). After soaking overnight in EtOAc, the solid medium was cut into small pieces and then shaken for 8 h at 150 rpm. The combined extracts were concentrated via evaporation of EtOAc under reduced pressure, yielding around 15 g of EtOAc crude extract. The crude extract (15 g) was fractionated through VLC (vacuum liquid chromatography) on silica gel as a stationary-phase with a gradient elution of solvents consisting of mixtures of *n*-hexane/EtOAc and CH_2_Cl_2_/MeOH, to afford seven fractions (A. ch Fr. 1–7). Fraction A. ch. Fr. 4 (640 mg), eluted with 100% EtOAc, was further purified via Sephadex LH20 column chromatography using CH_2_Cl_2_-MeOH (1:1) as a mobile phase, yielding three subfractions (A. ch Fr. 4.1–4.3). Subfraction (A. ch Fr. 4.2) (205.6 mg) was purified using semi-preparative HPLC with a gradient of MeOH-H_2_O containing 0.1% formic acid (65:35 to 95:05 in 21 min) to afford **1** (4.43 mg), **2** (1.04 mg), **3** (11.02 mg), **4** (40.48 mg), **5** (4.60 mg), **6** (3.01 mg), and **7** (1.47 mg) eluting at retention times; 33.5, 29.8, 25.7, 31.4, 32.0, 35.2, and 36.9 min, respectively

Rubrumline P (1): beige-brown residue; [*α*]^20^_*D*_-14.216 (*c* 0.10, CHCl_3_); UV (MeOH) λ_max_ 371.0, 341.0 and 210.8 nm; ^1^H and ^13^C NMR data, Table 1; HRESIMS *m/z* 508.3173 [M + H]^+^ (calcd for C_30_H_42_N_3_O_4_, 508.3170).

### 3.4. Crystallographic Analysis of Compound 2

Crystals were obtained through solvent evaporation (MeOH). The data collection process was as follows: Single crystals were measured on a Rigaku XtaLAB Synergy-S, Dualflex, HyPix diffractometer with a micro-focus X-ray tube, with Cu-Kα radiation (λ = 1.54182 Å) at 301.6(1) K. Cell refinement, data collection, and data reduction were performed with CrysalisPro [35]. The Structure solution was conducted using SHELXT 2014/4 [36]. Structure refinement was performed with SHELXL 2017/1 [37], and implemented in the Olex2 software (version 1.5) package [38]. All non-hydrogen atoms were refined with anisotropic displacement parameters. All hydrogen atoms on C were positioned geometrically (with C–H = 0.93 Å for aromatic and vinyl CH, 0.98 Å for aliphatic CH, 0.97 Å for CH_2_, and 0.96 Å for CH_3_) and refined using riding models (AFIX 43, 93, 13, 23, and 137 with U_iso(H)_ = 1.2 U_eq(C)_ (CH, CH_2_) and 1.5 U_eq(C)_ (CH_3_)). Protic hydrogen atoms on N and O were found and refined with U_iso(H)_ = 1.5 U_eq(N/O)_. The crystal structure was deposited into the Cambridge Crystallographic Data Center (CCDC no. 2308479).

### 3.5. DNA Isolation and Genome Sequencing

DNA isolation was performed according to the Quick DNA fungal/bacterial miniprep protocol provided by Zymo Research [39].

For long-read Nanopore sequencing, the NBE_9169_v114_revH_15Sep2022 protocol for ligation sequencing of genomic DNA was followed, using the SQK-NBD114 native barcoding kit and a PromethION Flow Cell R10 Version. Reads were assembled using Flye Version 2.9.2-b1794 [40] with asmCoverage-Mode. Antismash [41] and InterProScan Version 5.58-91.0 [42] were used to detect gene clusters and identify potential genes of interest.

### 3.6. Cell Culture and Cytotoxic Activity

The PDAC cell line PANC-1, purchased from the American Type Culture Collection (ATCC), was cultured in Dulbecco’s modified Eagle medium (DMEM) (#41965039, Gibco, Grand Island, NY, USA) supplemented with 10% fetal bovine serum (FBS) (#10270-106, Gibco, Grand Island, NY, USA), as well as 1% penicillin-streptomycin (#15140122, Gibco, Grand Island, NY, USA). Cells were incubated at 37 °C in a humidified atmosphere supplemented with 5% CO_2_. Cell viability was assessed using the PrestoBlue HS Cell Viability Assay (#P50201, Invitrogen^TM^, Waltham, MA, USA) in 96-well plates (cat. no. 655090, Greiner Bio-One, Frickenhausen, Germany) by seeding 5000 cells per well. Compounds were incubated for 72 h. Paclitaxel (#sc-201439, Santa Cruz Biotechnology, Heidelberg, Germany) dissolved in DMSO (#A994.1, Carl Roth, Karlsruhe, Germany) was used as a positive control. Fluorescence values were recorded using the Tecan SPARK instrument (Tecan Group, Männedorf, Switzerland), according to the manufacturer’s protocol.

### 3.7. Cell Cycle Analysis

For analysis of the cell cycles, 9 × 10^4^ cells/mL of PANC-1 were cultured in 6-well plates (Greiner Bio-One). After treatment for 24 h and harvesting via trypsin, the cells were washed with D-PBS. For cell cycle analyses, the cells were suspended in 100 µL of hypotonic buffer (1% sodium citrate, 0.1% Triton X-100, and 50 µg/mL propidium iodide in double-distilled water). The suspension was incubated for 10 min at room temperature. Flow cytometry was performed with the BD FACSLyric flow cytometer (#87135, BD Biosciences, Heidelberg, Germany), whereby FlowJo software (version 10.8.1) was utilized for the analysis.

### 3.8. Statistical Analysis

GraphPad Prism version 8.4.3 (GraphPad Software, California, USA) was used to perform statistical analyses and graphical illustrations. Non-linear regression was performed to assess cell viability, obtaining IC_50_ values. To determine statistical significance, a one-way analysis of variance (ANOVA) was used, wherein a *p*-value below 0.05 was defined as statistically significant.

## 4. Conclusions

Following a bioassay-guided strategy for the isolation of presumable drugs or drug-led structures originating from a natural source for use against the human pancreatic adenocarcinoma cell line PANC-1, several indole diketopiperazine alkaloids, including a new triprenylated alkaloid, named rubrumline P (**1**), together with other known prenylated derivatives (**2**–**7**) were obtained from rice culture supplemented with 3.5% NaCl of the marine sediment-derived fungus, *Aspergillus chevalieri*. Two isolated indole DKPs, rubrumline P (**1**) and neoechinulin D (**4**), showed cytotoxic efficacy with IC_50_ values of 25.8 and 23.4 µM, respectively. In addition, a cell cycle analysis of the isolated secondary metabolites has been conducted. Most notably, our analysis of the fungal genome of *A. chevalieri*, which is described for the first time, revealed the ability of this fungus to synthesize prenyltransferases as tailoring enzymes involved in the production of prenylated compounds, as evidenced by the isolation of different prenylated indole DKPs from the fungus. This represents a starting point for the optimization of such prenylated alkaloids for future drug discoveries and development.

## Figures and Tables

**Table 1:** ^1^H (600 MHz) and ^13^C (150 MHz) NMR spectroscopic data recorded in CDCl_3_ for compound **1** (*δ* in ppm).

No.	*δ*_C_, Type	*δ*_H_ (Mult, *J* in Hz)	HMBC (from H to C)
2	144.7, C		
3	102.8, C		
3a	126.6, C		
4	116.6, CH	6.93, d (1.5)	3, 6, 7a, 21
5	134.5, C		
6	123.8, CH	6.74, d (1.5)	4, 7a, 26
7	123.5, C		
7a	132.9, C		
8	113.2, CH	7.23, s	2, 3a, 10
9	124.0, C		
10	160.4, C		
12	51.8, CH	4.27, q (6.9)	10, 13, 20
13	165.5, C		
15	39.4, C		
16	144.6, CH	6.05, dd (17.4, 10.5)	2, 15, 18, 19
17	112.7, CH_2_	5.14, d (10.5)	15, 16
		5.12, d (17.4)	
18	27.6, CH_3_	1.51, s	2, 15, 16, 19
19	27.5, CH_3_	1.50, s	2, 15, 16, 18
20	21.1, CH_3_	1.60, d (6.9)	12, 13
21	36.3, CH_2_	2.85, d (15.0)	7, 7a, 28
		2.94, dd (15.0, 8.7)	
22	78.2, CH	3.80, d (8.7)	7, 26, 28, 29, 30
23	77.8, C		
24	20.5, CH_3_	1.28, s	27, 28, 30
25	18.5, CH_3_	1.23, s	27, 28, 29
26	34.6, CH_2_	3.38, m	4, 5, 6, 22, 23
27	124.3, CH	5.35, m	24, 25
28	132.0, C		
29	25.9, CH_3_	1.72, s	22, 23, 25
30	18.0, CH_3_	1.71, s	22, 23, 24
1-NH		10.21, s	2, 3, 3a, 7a
11-NH		6.34, br s	10, 13
14-NH		7.47, br s	10, 12, 13
23-OCH_3_	49.4, OCH_3_	3.28, s	28

**Table 2:** Length and coverage of the *Aspergillus chevalieri* genome contigs and the identified secondary metabolite regions.

Contig No.	Length (bp)	Coverage (x-Fold)	Secondary Metabolite Regions
2	4,649,651	4036	NRPS-like; fungal-RiPP-like; terpene; NI-siderophore
187	3,079,001	4045	T1PKS
123	2,864,395	4042	NRPS; T1PKS
260	1,732,854	4039	NRPS
71	1,589,188	4047	NRPS; fungal-RiPP-like; terpene
284	1,488,407	4039	NRPS-like
328	1,357,148	4052	no secondary metabolite region found
315	1,177,976	4057	no secondary metabolite region found
149	21,917	3994	Terpenoid cyclases/Protein prenyltransferases

**Table 3:** Cytotoxic efficacy in PANC-1 cells represented by the respective IC_50_ values after 72 h of incubation. The arithmetic means of three independent biological repeats and their respective standard deviations are depicted.

Compound	IC_50_ (±SD) [µM]
	PANC-1
**1**	25.8 ± 1.3
**4**	23.4 ± 1.8

## Data Availability

The CCDC number 2308479 for compound **2** contains the supplementary crystallographic data reported in this paper. These data can be obtained free of charge from the Cambridge Crystallographic Data Center via www.ccdc.cam.ac.uk/data_request/cif (accessed on 16 November 2023).

## Supplementary Materials

The following supporting information can be downloaded at: https://www.mdpi.com/article/10.3390/md22010005/s1, Figures S1–S10: The UV spectrum, HRESIMS, 1D and 2D NMR spectra of compound **1**, and the packing diagram, determined via single-crystal x-ray diffraction, and hydrogen bond network of compound **2**; Tables S1–S5: crystal data and refinement, fractional atomic coordinates, atomic displacement parameters, geometric parameters, and hydrogen bond geometry for compound **2.**
