# Supplementary material for: Indole Diketopiperazine Alkaloids from the Marine Sediment-Derived Fungus Aspergillus chevalieri against Pancreatic Ductal Adenocarcinoma
Source: Mar Drugs. 2023 Dec 20;22(1):5. doi: 10.3390/md22010005 (PMC10820104; doi:10.3390/md22010005)

Supplementary Material

Indole Diketopiperazine Alkaloids from the Marine Sediment-Derived Fungus *Aspergillus chevalieri* against Pancreatic Ductal Adenocarcinoma

Dina H. El-Kashef [1,2], Deborah D. Obidake [1], Katja Schiedlauske [1], Alina Deipenbrock [1], Sebastian Scharf [3], Hao Wang [4], Daniela Naumann [5], Daniel Friedrich [5], Simone Miljanovic [1], Takin Haj Hassani Sohi [6], Christoph Janiak [6], Klaus Pfeffer [3], Peter Proksch [1] and Nicole Teusch [1,*]

1 Institute of Pharmaceutical Biology and Biotechnology, Heinrich Heine University Dusseldorf, 40225 Dusseldorf, Germany; dihas102@hhu.de (D.H.E.-K.)
2 Department of Pharmacognosy, Faculty of Pharmacy, Minia University, 61519 Minia, Egypt
3 Institute of Medical Microbiology and Hospital Hygiene, Medical Faculty, Heinrich Heine University, 40225 Dusseldorf, Germany
4 Hainan Key Laboratory for Research and Development of Natural Products from Li Folk Medicine, Institute of Tropical Bioscience and Biotechnology, Chinese Academy of Tropical Agricultural Sciences, Haikou 571101, China
5 Department of Chemistry and Biochemistry, University of Cologne, 50939 Cologne, Germany
6 Institute of Inorganic Chemistry and Structural Chemistry, Heinrich Heine University Dusseldorf, 40225 Dusseldorf, Germany
* Correspondence: nicole.teusch@hhu.de; Tel.: +49-(0)-211-81-14163

Contents

Figure S1. UV spectrum of compound **1**. 2

Figure S2. HRESIMS of compound **1**. 3

Figure S3. ^{1}H NMR (600 MHz, $CDCl_3$) spectrum of compound **1**. 4

Figure S4. APT ^{13}C NMR (150 MHz, $CDCl_3$) spectrum of compound **1**. 5

Figure S5. COSY (600 MHz, $CDCl_3$) spectrum of compound **1**. 6

Figure S6. HSQC (600 MHz/150 MHz, $CDCl_3$) spectrum of compound **1**. 7

Figure S7. HMBC (600 MHz/150 MHz, $CDCl_3$) spectrum of compound **1**. 8

Figure S8. NOESY (600 MHz, $CDCl_3$) spectrum of compound **1**. 9
Table S1: Crystal data and refinement for compound **2**. 10
Table S2. Fractional atomic coordinates and isotropic or equivalent isotropic displacement parameters (Å^2) for Compound **2**. 11
Table S3. Atomic displacement parameters (Å^2) for compound **2**. 13
Table S4. Geometric parameters (Å, º) for compound **2**. 13
Table S5. Hydrogen-bond geometry (Å, º) for compound **2**. 17
Figure S9. a) Section of the packing diagram over the unit cell of compound **2** determined by single-crystal x-ray diffraction. b) Hydrogen bond network as in dashed yellow lines around a molecule of **2** in the crystal (the dimethylvinylmethyl or prenyl group, -$CMe_2(CH{=}CH_2)$ has been omitted for clarity). For details see Table S5 above. 18
Figure S10. X-ray crystallographic structure of compound **2** 19

Figure S1. UV spectrum of compound **1**.

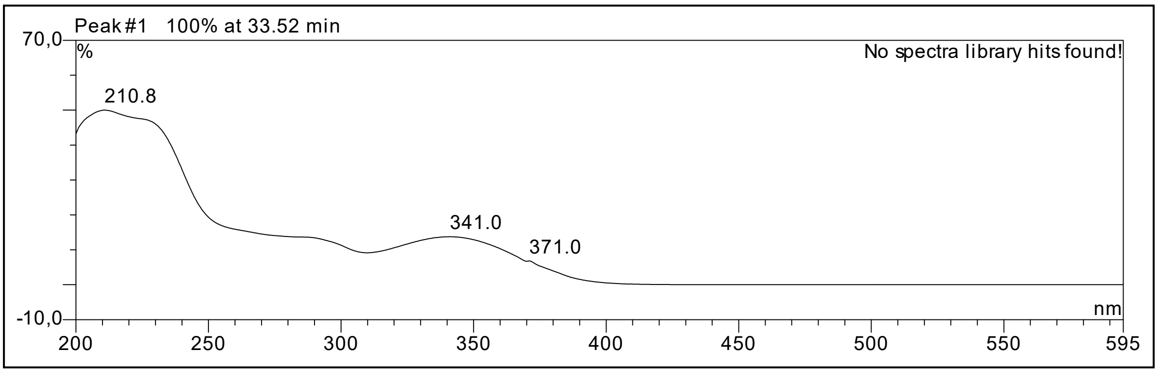

Figure S2. HRESIMS of compound **1**.

Mass Spectrum SmartFormula Report

Analysis Info

Analysis Name D:\Data\Spektren 2023\TEU23HR000061.d
Method tune_low_new.m
Sample Name El-Kashef A.Ch.fr. 7.2-P5 (CH3OH)
Comment

Acquisition Date 5/30/2023 10:34:01 AM

Operator PT
Instrument maXis 288882.20213

Acquisition Parameter

Source Type	ESI	Ion Polarity	Positive	Set Nebulizer	0.3 Bar
Focus	Not active	Set Capillary	4000 V	Set Dry Heater	180 °C
Scan Begin	50 m/z	Set End Plate Offset	-500 V	Set Dry Gas	4.0 l/min
Scan End	1500 m/z	Set Collision Cell RF	600.0 Vpp	Set Divert Valve	Source

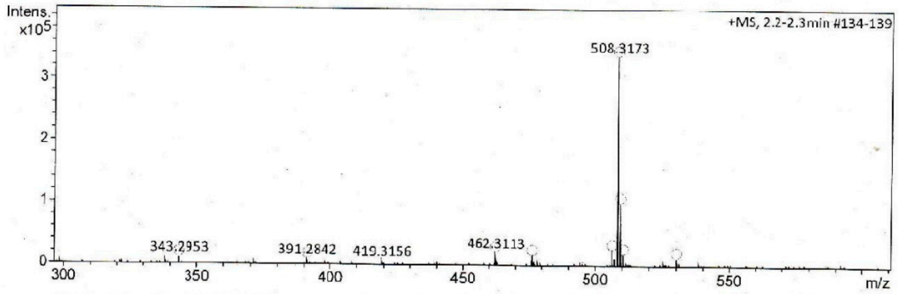

Meas. m/z	#	Ion Formula	m/z	err [ppm]	mSigma	# mSigma	Score	rdb	e⁻ Conf	N-Rule
343.2953	1	C19H39N2O3	343.2955	0.5	12.3	1	100.00	1.5	even	ok
391.2842	1	C25H35N4	391.2856	3.6	13.3	1	51.88	10.5	even	ok
	2	C24H39O4	391.2843	0.2	15.8	2	100.00	5.5	even	ok
462.3113	1	C29H40N3O2	462.3115	0.4	20.0	1	100.00	11.5	even	ok
476.2907	1	C29H38N3O3	476.2908	0.0	282.9	1	100.00	12.5	even	ok
506.3013	1	C31H36N7	506.3027	2.6	63.5	1	100.00	17.5	even	ok
	2	C18H44N5O11	506.3032	3.6	128.0	2	3.61	-0.5	even	ok
	3	C16H32N19O	506.3032	3.6	132.5	3	2.10	10.5	even	ok
	4	C15H36N15O5	506.3018	1.0	146.2	4	2.02	5.5	even	ok
	5	C14H40N11O9	506.3005	-1.7	159.9	5	0.67	0.5	even	ok
	6	C30H40N3O4	506.3013	-0.0	676.6	6	0.00	12.5	even	ok
508.3173	1	C30H42N3O4	508.3170	-0.6	22.9	1	100.00	11.5	even	ok
	2	C31H38N7	508.3183	2.0	34.3	2	52.17	16.5	even	ok
~~509.3203~~	~~1~~	~~C23H41N8O5~~	509.3194	-1.7	62.4	1	100.00	7.5	even	ok
	~~2~~	~~C27H45N2O7~~	509.3221	3.6	66.1	2	64.46	6.5	even	ok
	~~3~~	~~C24H37N12O~~	509.3208	0.9	75.7	3	75.56	12.5	even	ok
	~~4~~	~~C39H41~~	509.3203	-0.1	127.5	4	10.07	19.5	even	ok
510.3243	1	C20H44N7O8	510.3246	0.6	39.7	1	100.00	2.5	even	ok
	2	C17H36N17O2	510.3232	-2.1	40.9	2	65.83	8.5	even	ok
	3	C21H40N11O4	510.3259	3.2	53.3	3	31.49	7.5	even	ok
	4	C32H40N5O	510.3227	-3.0	98.8	4	7.45	15.5	even	ok
530.2987	1	C28H36N9O2	530.2986	-0.2	11.4	1	100.00	15.5	even	ok
	2	C27H40N5O6	530.2973	-2.7	14.3	2	46.68	10.5	even	ok

Figure S3. ^{1}H NMR (600 MHz, $CDCl_3$) spectrum of compound **1**.

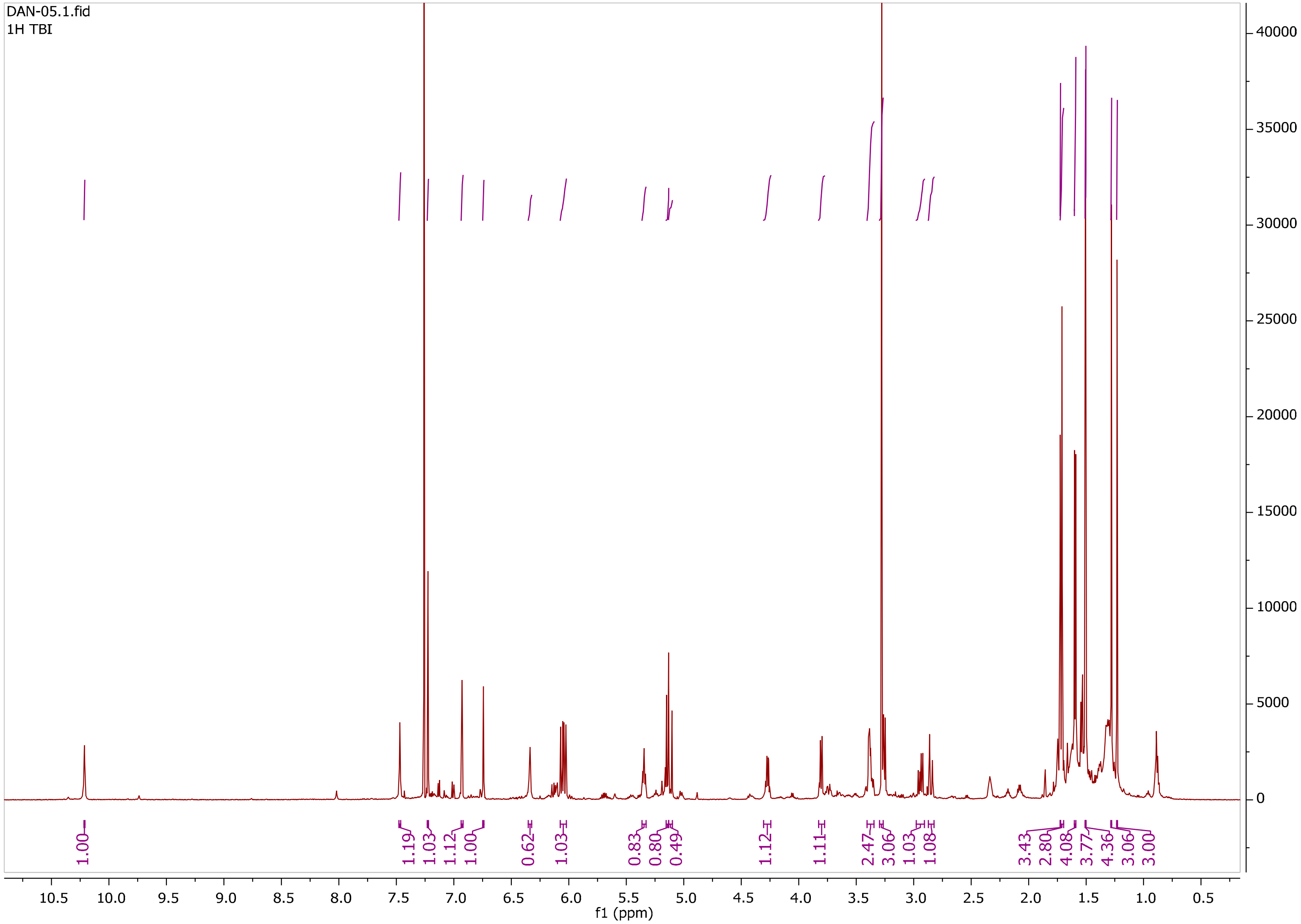

Figure S4. APT ^{13}C NMR (150 MHz, $CDCl_3$) spectrum of compound **1**.

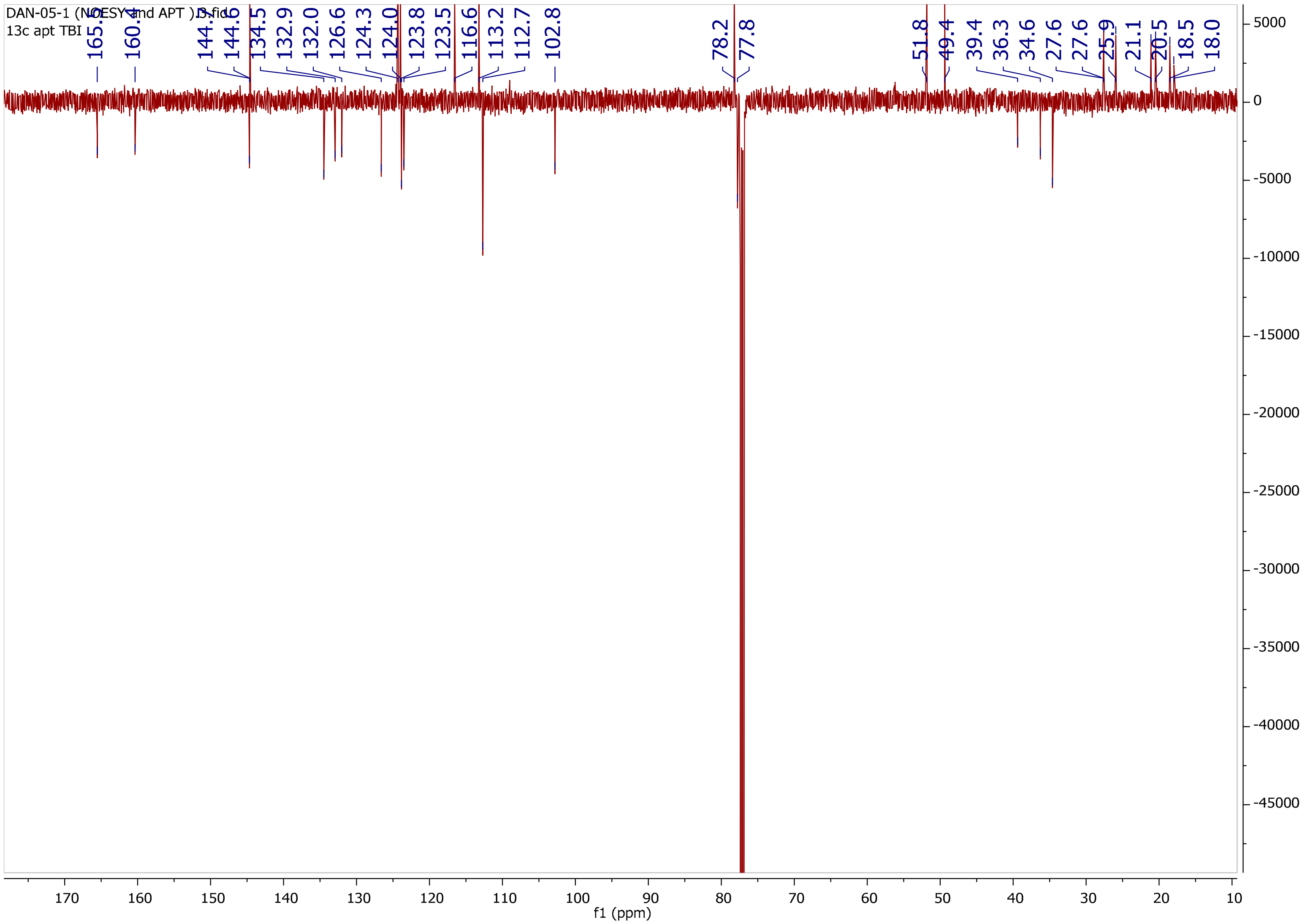

Figure S5. COSY (600 MHz, $CDCl_3$) spectrum of compound **1**.

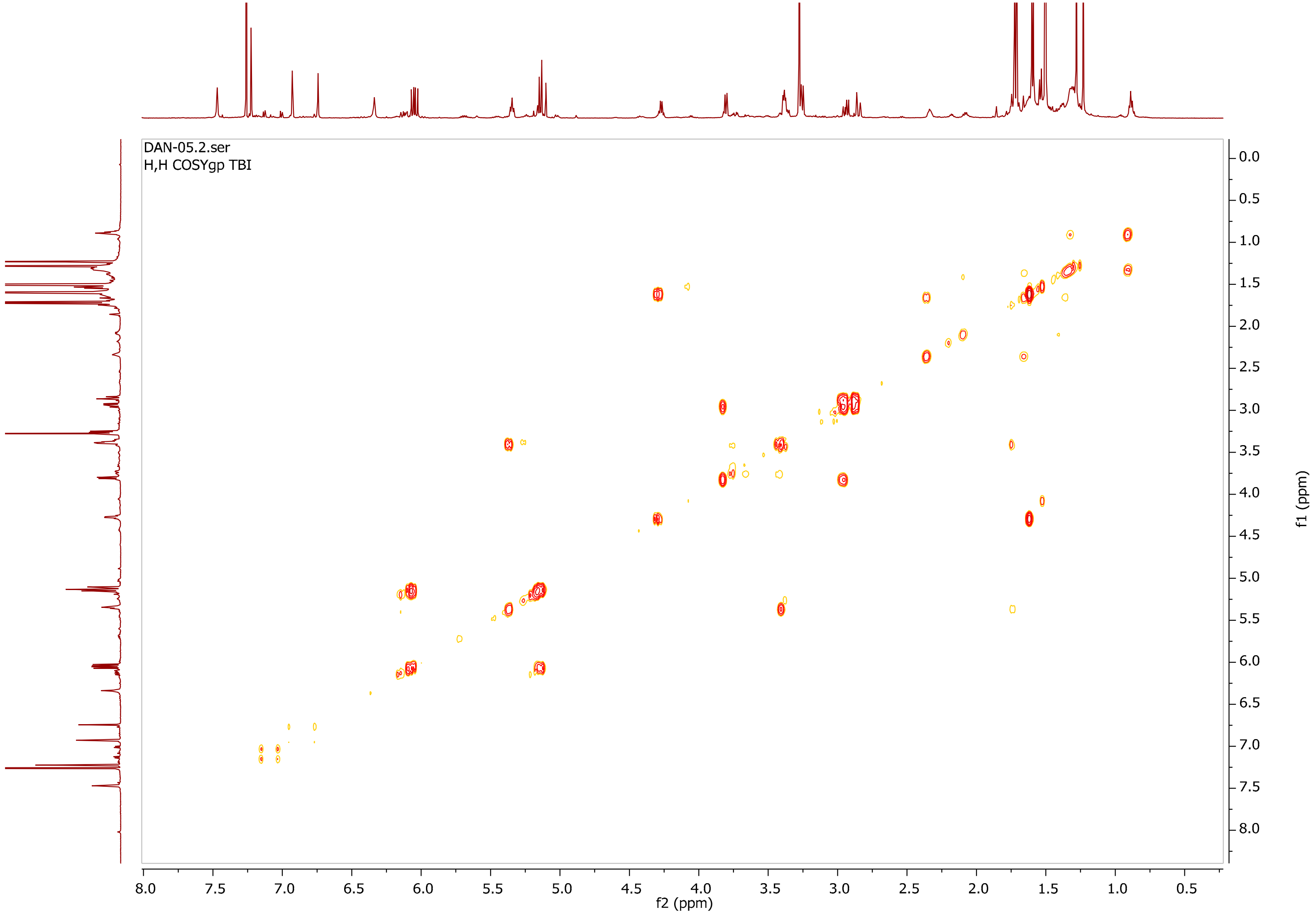

Figure S6. HSQC (600 MHz/150 MHz, $CDCl_3$) spectrum of compound **1**.

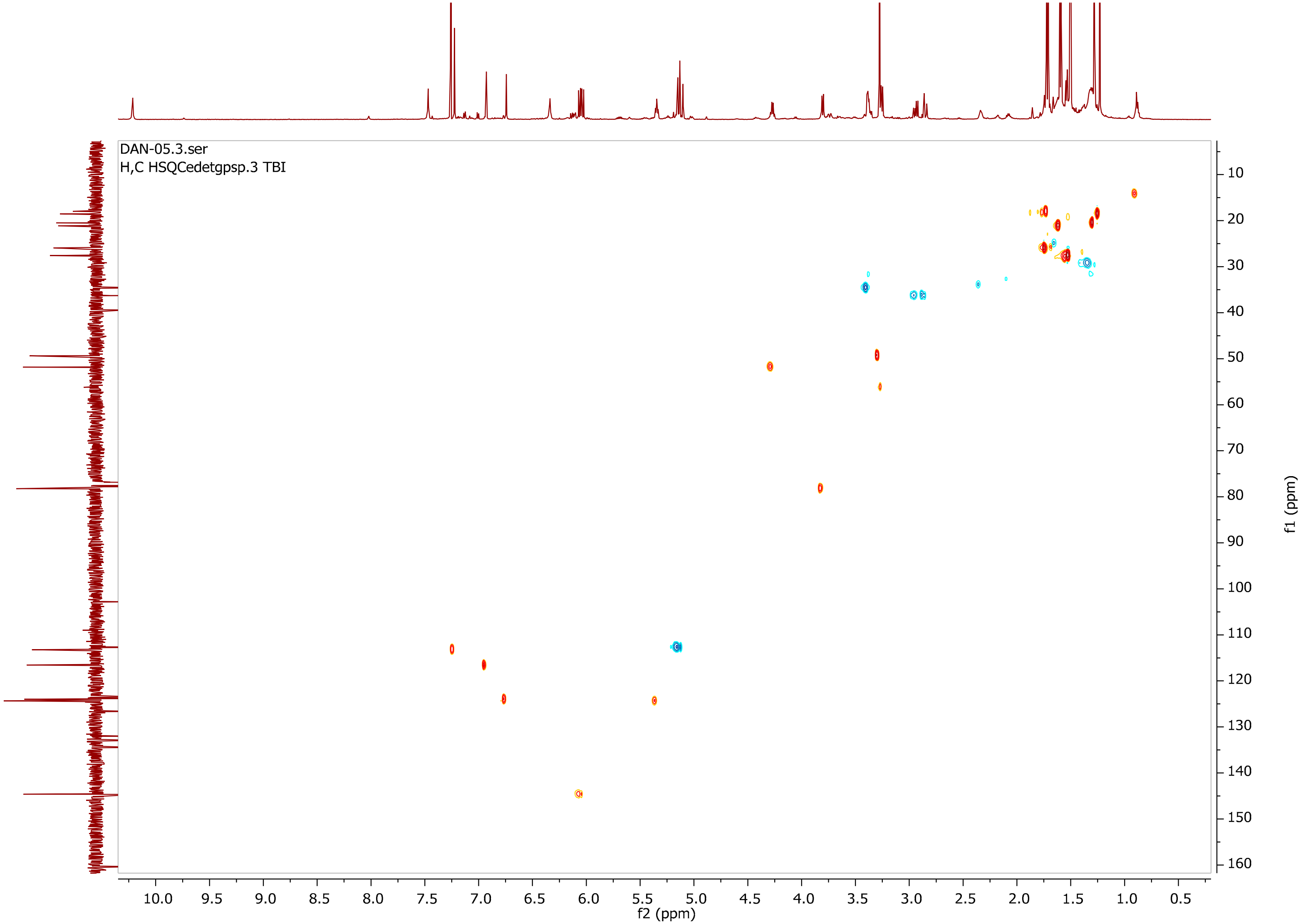

Figure S7. HMBC (600 MHz/150 MHz, $CDCl_3$) spectrum of compound **1**.

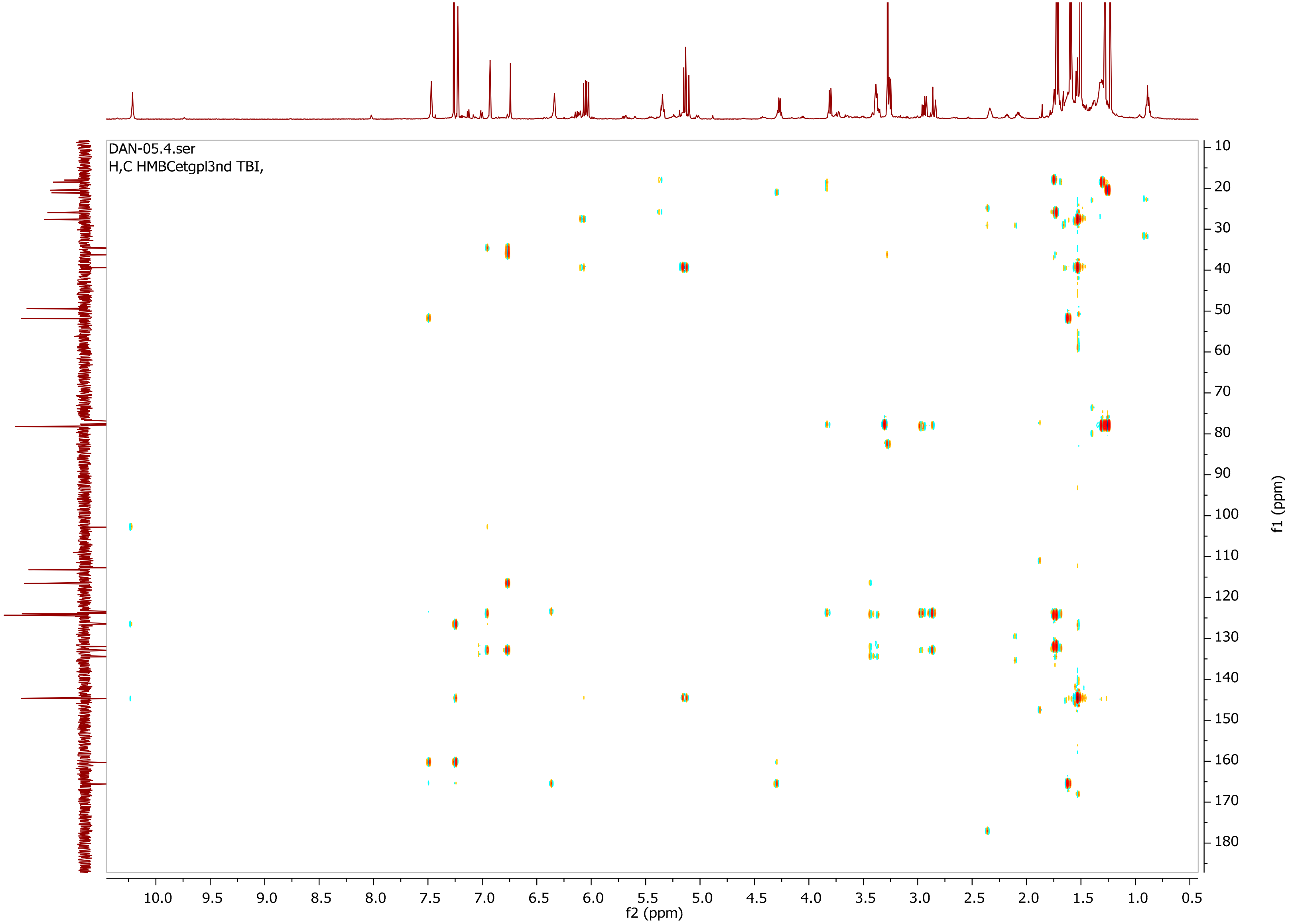

Figure S8. NOESY (600 MHz, $CDCl_3$) spectrum of compound **1**.

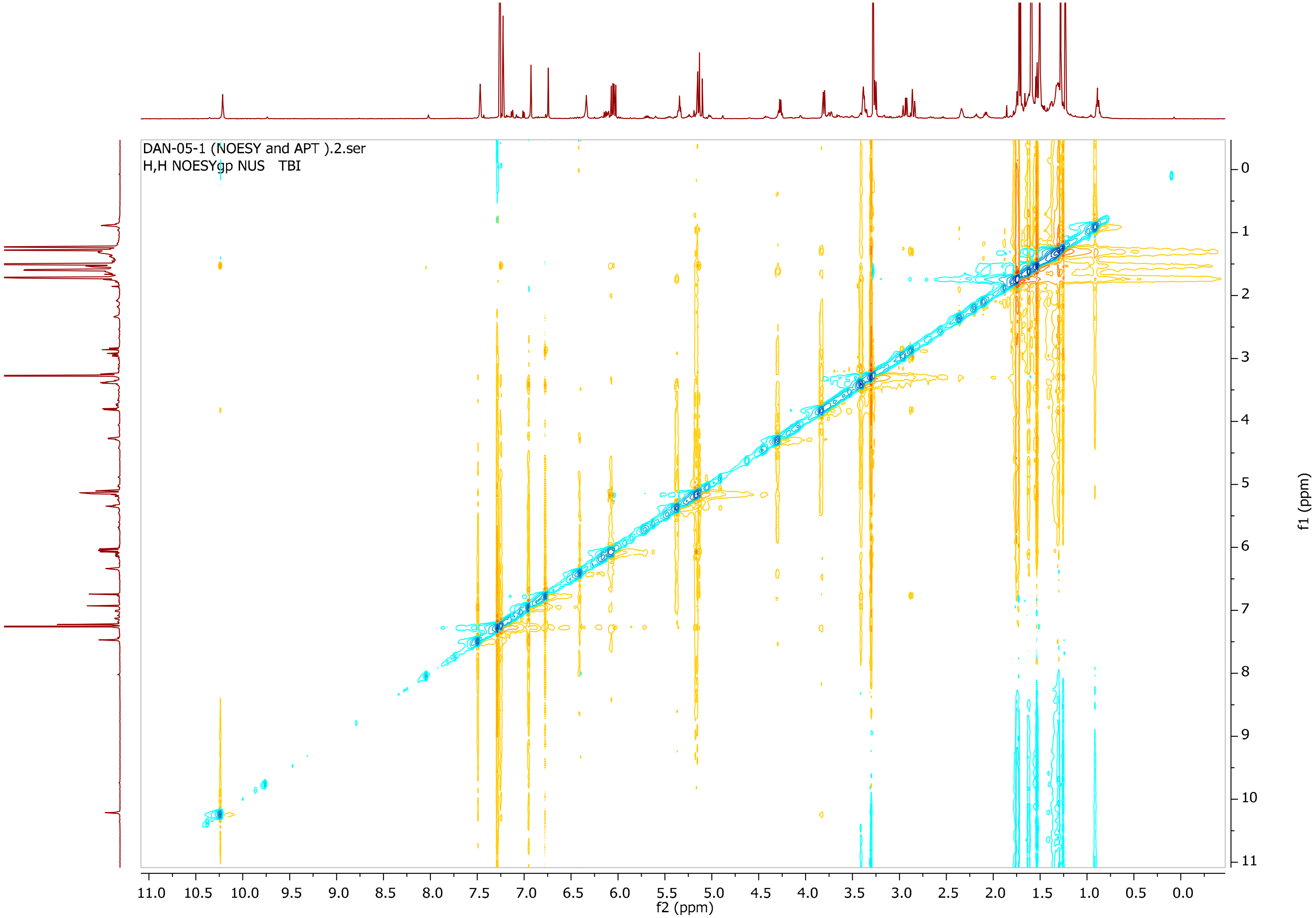

Table S1: Crystal data and refinement for compound **2**.

Crystal data

$C_{25}H_{33}N_3O_4$	D_x = 1.231 Mg m^{-3}
M_r = 439.54	Cu $K\alpha$ radiation, λ = 1.54184 Å
Orthorhombic, $P2_12_12_1$	Cell parameters from 6819 reflections
a = 6.0267 (1) Å	θ = 3.1–75.6°
b = 19.1922 (5) Å	μ = 0.68 mm^{-1}
c = 20.5108 (5) Å	T = 302 K
V = 2372.39 (9) Å^3	Needle, clear colourless
Z = 4	0.18 × 0.04 × 0.03 mm
F(000) = 944	**CCDC deposition no. 2308479**

Data collection

XtaLAB Synergy, Dualflex, HyPix diffractometer	4755 independent reflections
Radiation source: micro-focus sealed X-ray tube, PhotonJet (Cu) X-ray Source	3950 reflections with $I > 2\sigma(I)$
Mirror monochromator	R_{int} = 0.057
Detector resolution: 10.0000 pixels mm^{-1}	θ_{max} = 76.4°, θ_{min} = 3.2°
ω scans	h = -7→4
Absorption correction: analytical *CrysAlis PRO* 1.171.42.91a (Rigaku Oxford Diffraction, 2023) Analytical numeric absorption correction using a multifaceted crystal model based on expressions derived by R.C. Clark & J.S. Reid. (Clark, R. C. & Reid, J. S. (1995). Acta Cryst. A51, 887-897) Empirical absorption correction using spherical harmonics, implemented in SCALE3 ABSPACK scaling algorithm.	k = -23→24
T_{min} = 0.998, T_{max} = 0.999	l = -25→23
15025 measured reflections	

Refinement

Refinement on F^2	Hydrogen site location: mixed
Least-squares matrix: full	H atoms treated by a mixture of independent and constrained refinement
$R[F^2 > 2\sigma(F^2)]$ = 0.047	$w = 1/[\sigma^2(F_o^2) + (0.0847P)^2 + 0.0358P]$ where $P = (F_o^2 + 2F_c^2)/3$
$wR(F^2)$ = 0.132	$(\Delta/\sigma)_{max}$ < 0.001
S = 1.03	$\Delta\rangle_{max}$ = 0.21 e Å^{-3}
4755 reflections	$\Delta\rangle_{min}$ = -0.21 e Å^{-3}
308 parameters	Absolute structure: Flack x determined using 1419

	quotients [(I+)-(I-)]/[(I+)+(I-)] (Parsons, Flack and Wagner, Acta Cryst. B69 (2013) 249-259).
0 restraints	Absolute structure parameter: 0.08 (16)
Primary atom site location: dual	

Table S2. Fractional atomic coordinates and isotropic or equivalent isotropic displacement parameters (Å^2) for Compound **2**.

N1	0.6920 (4)	0.43010 (12)	0.64065 (11)	0.0452 (5)
O1	-0.1655 (3)	0.41347 (13)	0.82832 (11)	0.0626 (6)
C2	0.5057 (4)	0.45595 (15)	0.67234 (12)	0.0443 (6)
O2	0.5434 (3)	0.30001 (15)	0.93856 (12)	0.0711 (7)
C3	0.4274 (4)	0.40504 (15)	0.71386 (12)	0.0442 (6)
C3A	0.5705 (4)	0.34492 (14)	0.70642 (12)	0.0459 (6)
O3	0.9767 (6)	0.27362 (14)	0.49314 (13)	0.0797 (7)
C4	0.5747 (6)	0.27719 (17)	0.73192 (15)	0.0605 (8)
H4	0.465673	0.262414	0.760865	0.073*
O4	0.9752 (4)	0.46074 (13)	0.50915 (12)	0.0709 (6)
C5	0.7422 (6)	0.23311 (18)	0.71353 (16)	0.0693 (9)
H5	0.747372	0.188534	0.731249	0.083*
C6	0.9050 (6)	0.25306 (18)	0.66904 (15)	0.0626 (8)
H6	1.016957	0.221716	0.658385	0.075*
C7	0.9045 (5)	0.31848 (15)	0.64024 (13)	0.0485 (6)
C7A	0.7329 (4)	0.36268 (14)	0.66053 (12)	0.0446 (6)
C8	0.2323 (4)	0.40736 (15)	0.75631 (13)	0.0471 (6)
H8	0.105970	0.428071	0.738941	0.056*
C9	0.2151 (4)	0.38324 (15)	0.81686 (13)	0.0438 (6)
C10	-0.0029 (4)	0.38180 (16)	0.85016 (14)	0.0479 (6)
N11	-0.0142 (4)	0.34543 (15)	0.90600 (12)	0.0533 (6)
C12	0.1519 (4)	0.29439 (17)	0.92553 (14)	0.0504 (7)
H12	0.147277	0.290637	0.973158	0.060*
C13	0.3811 (4)	0.31885 (17)	0.90667 (14)	0.0505 (7)
N14	0.3975 (4)	0.35924 (14)	0.85399 (11)	0.0473 (5)
H14	0.527 (6)	0.373 (2)	0.8419 (17)	0.061 (10)*
H3	1.120 (9)	0.266 (3)	0.490 (2)	0.091*
C15	0.4423 (5)	0.53139 (16)	0.66059 (15)	0.0528 (7)
C19	0.1976 (6)	0.5450 (2)	0.6739 (3)	0.0896 (14)
H19A	0.108958	0.515081	0.646929	0.134*

H19B	0.163676	0.592767	0.664225	0.134*
H19C	0.165990	0.535749	0.718950	0.134*
C18	0.4861 (9)	0.5515 (2)	0.58863 (19)	0.0917 (14)
H18A	0.641868	0.547726	0.579513	0.138*
H18B	0.438418	0.598588	0.581280	0.138*
H18C	0.405119	0.520691	0.560468	0.138*
C16	0.5736 (6)	0.58073 (19)	0.7017 (2)	0.0713 (9)
H16	0.556226	0.627650	0.691577	0.086*
C17	0.7054 (8)	0.5679 (3)	0.7485 (2)	0.0887 (12)
H17A	0.730780	0.522052	0.761242	0.106*
H17B	0.776619	0.604219	0.769906	0.106*
C20	0.1033 (7)	0.2233 (2)	0.8978 (2)	0.0841 (12)
H20A	-0.039426	0.207851	0.912703	0.126*
H20B	0.215115	0.191041	0.911977	0.126*
H20C	0.103403	0.225702	0.851047	0.126*
C21	1.0640 (5)	0.34213 (16)	0.58915 (13)	0.0510 (6)
H21A	1.193512	0.312132	0.589677	0.061*
H21B	1.112382	0.389161	0.599022	0.061*
C22	0.9602 (5)	0.34074 (16)	0.52133 (14)	0.0536 (7)
H22	0.801375	0.349419	0.527633	0.064*
C23	1.0431 (5)	0.3981 (2)	0.47509 (15)	0.0618 (8)
C24	1.2949 (6)	0.3965 (2)	0.4668 (2)	0.0745 (10)
H24A	1.339837	0.433495	0.438213	0.112*
H24B	1.338340	0.352537	0.448359	0.112*
H24C	1.364693	0.402300	0.508472	0.112*
C25	0.9277 (8)	0.3909 (3)	0.40979 (18)	0.0964 (15)
H25A	0.769935	0.391959	0.416103	0.145*
H25B	0.969269	0.347521	0.389982	0.145*
H25C	0.970922	0.428745	0.381908	0.145*
C26	0.9967 (9)	0.5252 (3)	0.4747 (3)	0.1006 (15)
H26A	0.883027	0.528295	0.442090	0.151*
H26B	1.139797	0.527288	0.454283	0.151*
H26C	0.981694	0.563267	0.504783	0.151*
H1	0.769 (12)	0.452 (4)	0.607 (3)	0.151*
H11	-0.154 (13)	0.338 (4)	0.923 (3)	0.151*

Table S3. Atomic displacement parameters (Å^2) for compound **2**.

	U^{11}	U^{22}	U^{33}	U^{12}	U^{13}	U^{23}
N1	0.0447 (12)	0.0460 (12)	0.0450 (11)	0.0016 (9)	0.0073 (9)	0.0037 (9)
O1	0.0327 (10)	0.0846 (16)	0.0704 (13)	0.0065 (9)	0.0037 (8)	0.0218 (12)
C2	0.0402 (13)	0.0501 (14)	0.0427 (12)	0.0016 (11)	0.0019 (10)	0.0009 (11)
O2	0.0359 (10)	0.107 (2)	0.0707 (13)	0.0043 (10)	-0.0002 (9)	0.0359 (13)
C3	0.0394 (13)	0.0537 (14)	0.0394 (12)	-0.0007 (11)	-0.0009 (10)	0.0014 (11)
C3A	0.0489 (14)	0.0491 (14)	0.0396 (12)	0.0004 (11)	0.0020 (10)	0.0044 (11)
O3	0.0989 (18)	0.0689 (16)	0.0714 (14)	-0.0142 (14)	0.0113 (14)	-0.0217 (13)
C4	0.075 (2)	0.0563 (17)	0.0500 (15)	0.0014 (15)	0.0130 (14)	0.0083 (13)
O4	0.0770 (15)	0.0627 (14)	0.0729 (14)	0.0099 (12)	0.0146 (12)	0.0180 (12)
C5	0.097 (3)	0.0508 (17)	0.0601 (17)	0.0110 (16)	0.0139 (18)	0.0136 (14)
C6	0.078 (2)	0.0554 (18)	0.0546 (16)	0.0176 (15)	0.0080 (15)	0.0044 (13)
C7	0.0507 (14)	0.0512 (16)	0.0435 (13)	0.0046 (12)	-0.0007 (11)	0.0004 (12)
C7A	0.0462 (14)	0.0469 (14)	0.0407 (12)	0.0012 (11)	0.0026 (10)	0.0025 (10)
C8	0.0364 (12)	0.0579 (16)	0.0469 (14)	-0.0013 (11)	-0.0010 (10)	0.0013 (12)
C9	0.0306 (12)	0.0551 (15)	0.0456 (14)	-0.0014 (10)	-0.0007 (10)	-0.0003 (11)
C10	0.0324 (12)	0.0612 (16)	0.0501 (14)	-0.0017 (11)	0.0004 (10)	0.0019 (12)
N11	0.0312 (11)	0.0767 (17)	0.0520 (12)	0.0040 (10)	0.0080 (9)	0.0105 (12)
C12	0.0361 (13)	0.0669 (19)	0.0482 (14)	-0.0004 (11)	0.0034 (10)	0.0081 (13)
C13	0.0348 (13)	0.0686 (19)	0.0480 (14)	0.0026 (11)	0.0013 (10)	0.0052 (13)
N14	0.0281 (10)	0.0706 (16)	0.0433 (11)	-0.0013 (9)	0.0037 (8)	0.0067 (11)
C15	0.0450 (15)	0.0494 (15)	0.0641 (16)	0.0043 (11)	0.0030 (12)	0.0023 (13)
C19	0.0466 (18)	0.060 (2)	0.162 (4)	0.0086 (15)	0.003 (2)	0.022 (2)
C18	0.140 (4)	0.057 (2)	0.077 (2)	0.021 (2)	0.008 (3)	0.0134 (18)
C16	0.070 (2)	0.062 (2)	0.083 (2)	0.0018 (16)	0.0110 (19)	-0.0110 (18)
C17	0.090 (3)	0.095 (3)	0.080 (3)	-0.007 (2)	0.002 (2)	-0.017 (2)
C20	0.071 (2)	0.068 (2)	0.113 (3)	-0.0082 (18)	0.017 (2)	-0.002 (2)
C21	0.0456 (15)	0.0564 (16)	0.0512 (14)	0.0073 (12)	0.0043 (12)	-0.0006 (12)
C22	0.0491 (15)	0.0612 (18)	0.0504 (14)	-0.0054 (12)	0.0051 (12)	-0.0063 (13)
C23	0.0575 (17)	0.078 (2)	0.0497 (15)	-0.0002 (15)	0.0086 (13)	0.0055 (14)
C24	0.0594 (19)	0.083 (2)	0.082 (2)	-0.0029 (18)	0.0231 (17)	0.0072 (19)
C25	0.091 (3)	0.148 (5)	0.0502 (19)	-0.010 (3)	0.0010 (18)	0.018 (2)
C26	0.103 (3)	0.085 (3)	0.114 (3)	0.011 (2)	0.006 (3)	0.045 (3)

Table S4. Geometric parameters (Å, º) for compound **2**.

N1—C2	1.389 (3)	N14—H14	0.86 (4)

N1—C7A	1.379 (4)	C15—C19	1.522 (4)
N1—H1	0.92 (7)	C15—C18	1.548 (5)
O1—C10	1.237 (3)	C15—C16	1.495 (5)
C2—C3	1.379 (4)	C19—H19A	0.9600
C2—C15	1.517 (4)	C19—H19B	0.9600
O2—C13	1.231 (3)	C19—H19C	0.9600
C3—C3A	1.449 (4)	C18—H18A	0.9600
C3—C8	1.463 (4)	C18—H18B	0.9600
C3A—C4	1.401 (4)	C18—H18C	0.9600
C3A—C7A	1.400 (4)	C16—H16	0.9300
O3—H3	0.88 (5)	C16—C17	1.269 (6)
O3—C22	1.415 (4)	C17—H17A	0.9300
C4—H4	0.9300	C17—H17B	0.9300
C4—C5	1.370 (5)	C20—H20A	0.9600
O4—C23	1.449 (4)	C20—H20B	0.9600
O4—C26	1.430 (5)	C20—H20C	0.9600
C5—H5	0.9300	C21—H21A	0.9700
C5—C6	1.394 (5)	C21—H21B	0.9700
C6—H6	0.9300	C21—C22	1.525 (4)
C6—C7	1.388 (4)	C22—H22	0.9800
C7—C7A	1.401 (4)	C22—C23	1.537 (4)
C7—C21	1.493 (4)	C23—C24	1.527 (5)
C8—H8	0.9300	C23—C25	1.515 (5)
C8—C9	1.329 (4)	C24—H24A	0.9600
C9—C10	1.481 (4)	C24—H24B	0.9600
C9—N14	1.415 (3)	C24—H24C	0.9600
C10—N11	1.343 (4)	C25—H25A	0.9600
N11—C12	1.457 (4)	C25—H25B	0.9600
N11—H11	0.92 (8)	C25—H25C	0.9600
C12—H12	0.9800	C26—H26A	0.9600
C12—C13	1.510 (4)	C26—H26B	0.9600
C12—C20	1.507 (5)	C26—H26C	0.9600
C13—N14	1.333 (4)		
C2—N1—H1	126 (4)	C15—C19—H19B	109.5
C7A—N1—C2	110.0 (2)	C15—C19—H19C	109.5
C7A—N1—H1	123 (4)	H19A—C19—H19B	109.5

N1—C2—C15	118.0 (2)	H19A—C19—H19C	109.5
C3—C2—N1	108.2 (2)	H19B—C19—H19C	109.5
C3—C2—C15	133.5 (2)	C15—C18—H18A	109.5
C2—C3—C3A	107.2 (2)	C15—C18—H18B	109.5
C2—C3—C8	128.4 (3)	C15—C18—H18C	109.5
C3A—C3—C8	124.4 (2)	H18A—C18—H18B	109.5
C4—C3A—C3	135.3 (3)	H18A—C18—H18C	109.5
C7A—C3A—C3	107.0 (2)	H18B—C18—H18C	109.5
C7A—C3A—C4	117.6 (3)	C15—C16—H16	115.3
C22—O3—H3	104 (3)	C17—C16—C15	129.3 (4)
C3A—C4—H4	120.5	C17—C16—H16	115.3
C5—C4—C3A	118.9 (3)	C16—C17—H17A	120.0
C5—C4—H4	120.5	C16—C17—H17B	120.0
C26—O4—C23	117.0 (3)	H17A—C17—H17B	120.0
C4—C5—H5	119.0	C12—C20—H20A	109.5
C4—C5—C6	122.0 (3)	C12—C20—H20B	109.5
C6—C5—H5	119.0	C12—C20—H20C	109.5
C5—C6—H6	119.1	H20A—C20—H20B	109.5
C7—C6—C5	121.7 (3)	H20A—C20—H20C	109.5
C7—C6—H6	119.1	H20B—C20—H20C	109.5
C6—C7—C7A	115.0 (3)	C7—C21—H21A	109.3
C6—C7—C21	124.9 (3)	C7—C21—H21B	109.3
C7A—C7—C21	120.0 (3)	C7—C21—C22	111.8 (2)
N1—C7A—C3A	107.6 (2)	H21A—C21—H21B	107.9
N1—C7A—C7	127.8 (2)	C22—C21—H21A	109.3
C3A—C7A—C7	124.6 (3)	C22—C21—H21B	109.3
C3—C8—H8	116.3	O3—C22—C21	111.1 (3)
C9—C8—C3	127.4 (2)	O3—C22—H22	106.1
C9—C8—H8	116.3	O3—C22—C23	112.2 (3)
C8—C9—C10	120.5 (2)	C21—C22—H22	106.1
C8—C9—N14	123.7 (2)	C21—C22—C23	114.6 (3)
N14—C9—C10	115.8 (2)	C23—C22—H22	106.1
O1—C10—C9	121.8 (2)	O4—C23—C22	101.8 (2)
O1—C10—N11	121.6 (2)	O4—C23—C24	110.6 (3)
N11—C10—C9	116.6 (2)	O4—C23—C25	111.8 (3)
C10—N11—C12	123.3 (2)	C24—C23—C22	112.2 (3)
C10—N11—H11	116 (4)	C25—C23—C22	109.3 (3)

C12—N11—H11	115 (4)	C25—C23—C24	110.8 (3)
N11—C12—H12	107.7	C23—C24—H24A	109.5
N11—C12—C13	110.4 (2)	C23—C24—H24B	109.5
N11—C12—C20	111.8 (3)	C23—C24—H24C	109.5
C13—C12—H12	107.7	H24A—C24—H24B	109.5
C20—C12—H12	107.7	H24A—C24—H24C	109.5
C20—C12—C13	111.3 (3)	H24B—C24—H24C	109.5
O2—C13—C12	120.0 (2)	C23—C25—H25A	109.5
O2—C13—N14	122.9 (3)	C23—C25—H25B	109.5
N14—C13—C12	117.1 (2)	C23—C25—H25C	109.5
C9—N14—H14	117 (2)	H25A—C25—H25B	109.5
C13—N14—C9	124.6 (2)	H25A—C25—H25C	109.5
C13—N14—H14	119 (2)	H25B—C25—H25C	109.5
C2—C15—C19	112.3 (3)	O4—C26—H26A	109.5
C2—C15—C18	110.3 (3)	O4—C26—H26B	109.5
C19—C15—C18	107.0 (3)	O4—C26—H26C	109.5
C16—C15—C2	112.5 (3)	H26A—C26—H26B	109.5
C16—C15—C19	107.6 (3)	H26A—C26—H26C	109.5
C16—C15—C18	106.8 (3)	H26B—C26—H26C	109.5
C15—C19—H19A	109.5		
N1—C2—C3—C3A	-0.9 (3)	C7—C21—C22—O3	-84.8 (3)
N1—C2—C3—C8	-178.5 (2)	C7—C21—C22—C23	146.7 (3)
N1—C2—C15—C19	156.7 (3)	C7A—N1—C2—C3	0.9 (3)
N1—C2—C15—C18	37.4 (4)	C7A—N1—C2—C15	175.8 (2)
N1—C2—C15—C16	-81.7 (3)	C7A—C3A—C4—C5	3.2 (5)
O1—C10—N11—C12	164.7 (3)	C7A—C7—C21—C22	-75.1 (3)
C2—N1—C7A—C3A	-0.5 (3)	C8—C3—C3A—C4	0.9 (5)
C2—N1—C7A—C7	-180.0 (3)	C8—C3—C3A—C7A	178.3 (2)
C2—C3—C3A—C4	-176.8 (3)	C8—C9—C10—O1	-13.0 (5)
C2—C3—C3A—C7A	0.6 (3)	C8—C9—C10—N11	168.2 (3)
C2—C3—C8—C9	-139.4 (3)	C8—C9—N14—C13	-161.7 (3)
C2—C15—C16—C17	-9.1 (5)	C9—C10—N11—C12	-16.6 (4)
O2—C13—N14—C9	-178.6 (3)	C10—C9—N14—C13	19.8 (4)
C3—C2—C15—C19	-30.0 (5)	C10—N11—C12—C13	37.8 (4)
C3—C2—C15—C18	-149.3 (3)	C10—N11—C12—C20	-86.6 (4)
C3—C2—C15—C16	91.6 (4)	N11—C12—C13—O2	151.8 (3)

C3—C3A—C4—C5	-179.6 (3)	N11—C12—C13—N14	-30.0 (4)
C3—C3A—C7A—N1	-0.1 (3)	C12—C13—N14—C9	3.2 (4)
C3—C3A—C7A—C7	179.4 (3)	N14—C9—C10—O1	165.6 (3)
C3—C8—C9—C10	-172.3 (3)	N14—C9—C10—N11	-13.1 (4)
C3—C8—C9—N14	9.2 (5)	C15—C2—C3—C3A	-174.7 (3)
C3A—C3—C8—C9	43.4 (4)	C15—C2—C3—C8	7.7 (5)
C3A—C4—C5—C6	-1.6 (5)	C19—C15—C16—C17	115.1 (5)
O3—C22—C23—O4	170.6 (3)	C18—C15—C16—C17	-130.3 (4)
O3—C22—C23—C24	-71.2 (4)	C20—C12—C13—O2	-83.4 (4)
O3—C22—C23—C25	52.1 (4)	C20—C12—C13—N14	94.8 (4)
C4—C3A—C7A—N1	177.8 (3)	C21—C7—C7A—N1	-2.8 (4)
C4—C3A—C7A—C7	-2.7 (4)	C21—C7—C7A—C3A	177.8 (3)
C4—C5—C6—C7	-0.9 (6)	C21—C22—C23—O4	-61.6 (3)
C5—C6—C7—C7A	1.6 (5)	C21—C22—C23—C24	56.7 (4)
C5—C6—C7—C21	-175.9 (3)	C21—C22—C23—C25	180.0 (3)
C6—C7—C7A—N1	179.7 (3)	C26—O4—C23—C22	-170.8 (3)
C6—C7—C7A—C3A	0.2 (4)	C26—O4—C23—C24	69.9 (4)
C6—C7—C21—C22	102.2 (4)	C26—O4—C23—C25	-54.1 (4)

Table S5. Hydrogen-bond geometry (Å, º) for compound **2**.

Nr Donor --- H....Acceptor [ARU]	D - H	H...A	D...A	D - H...A
1 Intra 1 N1--H1 O4 [ ]	0.93(7)	2.37(6)	3.246(3)	157(6)
2 1 O3--H3 O3 [3556.01]	0.88(5)	2.31(5)	3.159(5)	164(4)
3 1 N11--H11 O2 [1455.01]	0.92(8)	1.99(8)	2.884(3)	163(7)
4 1 N14--H14 O1 [1655.01]	0.86(4)	2.03(4)	2.880(3)	170(3)
5 Intra 1 C8--H8 O1 [ ]	0.93	2.47	2.818(3)	102
6 1 C12--H12 O2 [3457.01]	0.98	2.59	3.388(4)	139
7 1 C17--H17A O1 [1655.01]	0.93	2.57	3.474(6)	163
8 Intra 1 C21--H21B O4 [ ]	0.97	2.44	2.857(4)	105

Translation of ARU-Code to CIF and Equivalent Position Code

[1455.] = ii = –1+x, y, z
[1655.] = i = 1+x, y, z
[3556.] = iii = 1/2+x, 1/2–y, 1–z
[3457.] = –1/2+x, 1/2–y, 2–z

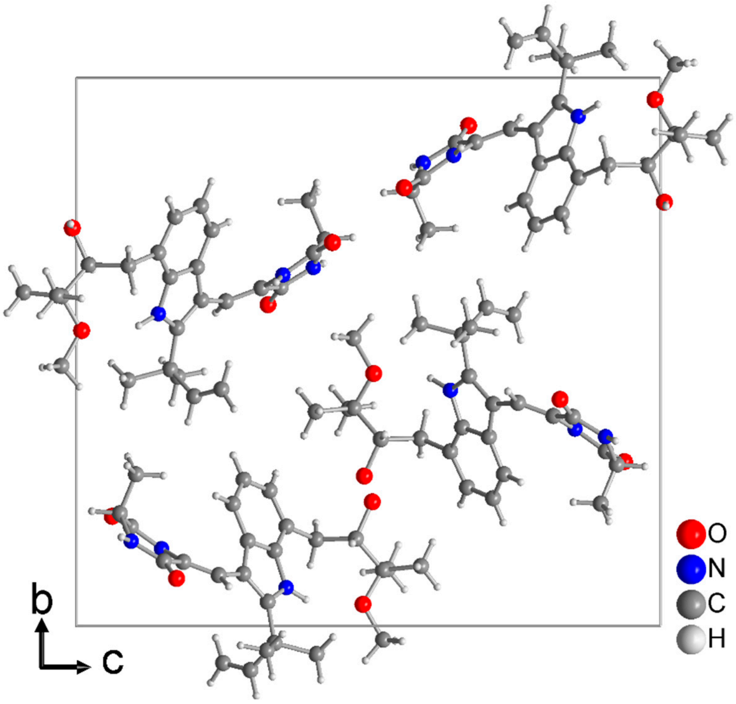

a)

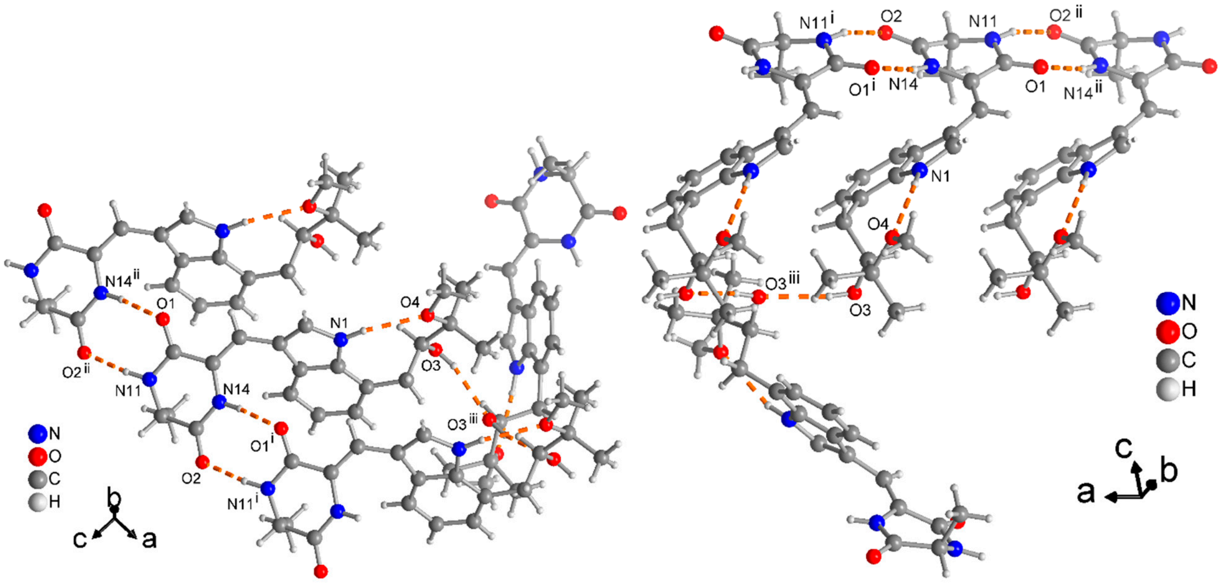

b)

Figure S9. a) Section of the packing diagram over the unit cell of compound **2** determined by single-crystal x-ray diffraction. b) Hydrogen bond network as in dashed yellow lines around a molecule of **2** in the crystal (the dimethylvinylmethyl or prenyl group, -CMe$_2$(CH=CH$_2$) has been omitted for clarity). For details see Table S5 above.

Figure S10. X-ray crystallographic structure of compound **2**

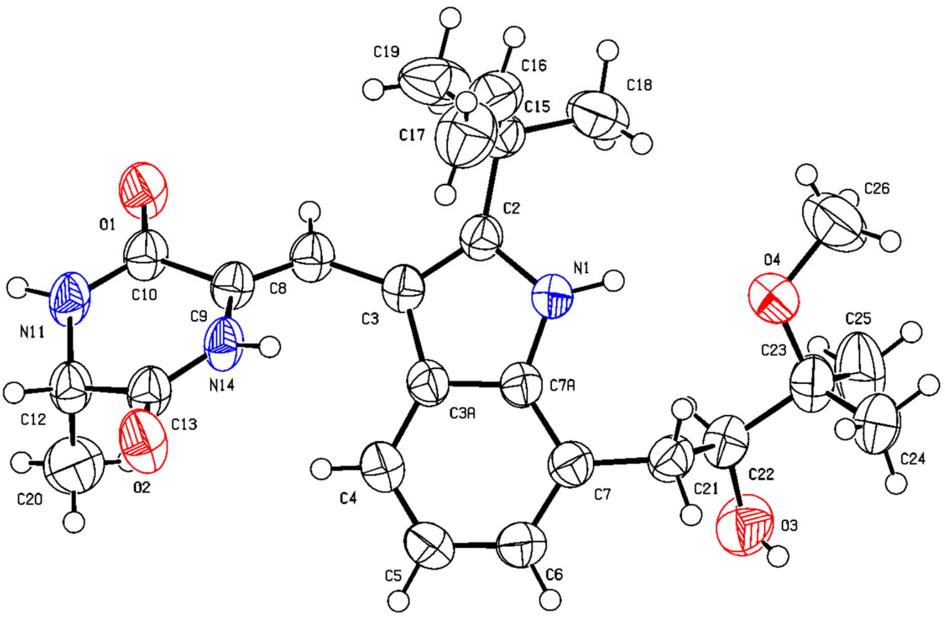

Supplement: Supplementary file 1 [file marinedrugs-22-00005-s001.zip › marinedrugs-2776028-supplementary.pdf]
